# Cortical reorganization after motor stroke: A pilot study on differences between the upper and lower limbs

**DOI:** 10.1002/hbm.25275

**Published:** 2020-11-09

**Authors:** Ellen Binder, Martha Leimbach, Eva‐Maria Pool, Lukas J. Volz, Simon B. Eickhoff, Gereon R. Fink, Christian Grefkes

**Affiliations:** ^1^ Department of Neurology, Faculty of Medicine and University Hospital Cologne University of Cologne Cologne Germany; ^2^ Institute of Neuroscience and Medicine (INM‐1, INM‐3) Research Centre Juelich Juelich Germany; ^3^ Department of Psychological and Brain Sciences University of California Santa Barbara California USA; ^4^ Institute for Clinical Neuroscience Heinrich‐Heine‐University Duesseldorf Germany

**Keywords:** dynamic causal modeling, effective connectivity, fMRI, interhemispheric inhibition, motor recovery, plasticity, rehabilitation

## Abstract

Stroke patients suffering from hemiparesis may show substantial recovery in the first months poststroke due to neural reorganization. While reorganization driving improvement of upper hand motor function has been frequently investigated, much less is known about the changes underlying recovery of lower limb function. We, therefore, investigated neural network dynamics giving rise to movements of both the hands and feet in 12 well‐recovered left‐hemispheric chronic stroke patients and 12 healthy participants using a functional magnetic resonance imaging sparse sampling design and dynamic causal modeling (DCM). We found that the level of neural activity underlying movements of the affected right hand and foot positively correlated with residual motor impairment, in both ipsilesional and contralesional premotor as well as left primary motor (M1) regions. Furthermore, M1 representations of the affected limb showed significantly stronger increase in BOLD activity compared to healthy controls and compared to the respective other limb. DCM revealed reduced endogenous connectivity of M1 of both limbs in patients compared to controls. However, when testing for the specific effect of movement on interregional connectivity, interhemispheric inhibition of the contralesional M1 during movements of the affected hand was not detected in patients whereas no differences in condition‐dependent connectivity were found for foot movements compared to controls. In contrast, both groups featured positive interhemispheric M1 coupling, that is, facilitation of neural activity, mediating movements of the affected foot. These exploratory findings help to explain why functional recovery of the upper and lower limbs often develops differently after stroke, supporting limb‐specific rehabilitative strategies.

## Graphical abstract

The present pilot study aimed to gain insights into the neural reorganization patterns of lower limb motor function in comparison to upper limb function in well‐recovered chronic stroke patients compared to healthy participants using functional magnetic resonance imaging and dynamic causal modeling. For the first time, we thus investigated unilateral movements of both the hands and feet in one common paradigm. This enabled direct comparisons of the respective reorganization patterns underlying hand and foot movements as well as differential conclusions regarding their functional relevance.

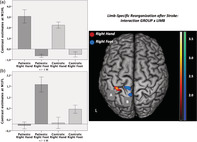

## 1 INTRODUCTION

Functional recovery after motor stroke is tightly linked to reorganizational processes within the motor system in both the ipsilesional and contralesional hemisphere (Cirillo et al., 2020; Grefkes & Ward, 2014). Numerous functional neuroimaging studies have reported increased neural activity in a bilateral network during movements of the stroke‐affected hand (e.g., Grefkes, Eickhoff, Nowak, Dafotakis, & Fink, 2008; Lotze et al., 2012; Ward, Brown, Thompson, & Frackowiak, 2003; Weiller, Chollet, Friston, Wise, & Frackowiak, 1992). A consistent finding across studies is that, compared to healthy controls, patients with chronic motor deficits often feature enhanced activity especially in the contralesional primary motor cortex (M1), bilateral ventral and dorsal premotor cortex (PMv, PMd), and the supplementary motor area (SMA) (Bestmann et al., 2010; Harrington et al., 2020; Rehme, Eickhoff, Rottschy, Fink, & Grefkes, 2012). Despite increased levels of neural activity, task‐dependent connectivity between premotor regions and the primary motor cortex is usually attenuated within the lesioned hemisphere, especially between ipsilesional SMA and M1 (Bajaj, Butler, Drake, & Dhamala, 2015a, 2015b; Bajaj et al., 2016; Grefkes, Nowak, et al., 2008; Sharma, Baron, & Rowe, 2009; Wang et al., 2016). However, the functional underpinnings of such neural changes concerning motor performance and recovery remain to be elucidated. With respect to hand motor performance, longitudinal functional magnetic resonance imaging (fMRI) studies have suggested a relationship of enhanced activity with the time poststroke, the degree of motor impairment, and the nature of the motor task performed during the scan (Cirillo et al., 2020; Rehme et al., 2012): Bilaterally increased neural activity within the motor network may play a supportive role early after stroke (Favre et al., 2014; Rehme, Fink, von Cramon, & Grefkes, 2011) and tends to resolve over time into lateralized activity patterns, especially in patients showing complete recovery of function (Calautti, Leroy, Guincestre, & Baron, 2001; Loubinoux et al., 2007; Nelles, Jentzen, Bockisch, & Diener, 2011; Saur et al., 2006; Ward et al., 2003). In contrast, patients suffering from severe, chronic motor impairments typically exhibit higher levels of neural activity, especially in the contralesional motor regions (Calautti et al., 2007; Loubinoux, 2003; Ward et al., 2003). Likewise, connectivity studies revealed that those patients with stronger increases of effective connectivity between ipsilesional motor regions show stronger recovery of hand motor function, while those patients developing inhibitory influences originating from the contralesional “healthy” hemisphere exhibit less successful recovery (Grefkes, Nowak, et al., 2008; Peters et al., 2018; Rehme, Fink, et al., 2011).

In contrast to the rich literature dealing with the neural mechanisms underlying the recovery of hand motor function, the reorganizational processes driving functional recovery of the lower limbs are less well understood. From a conceptual point of view, it appears very likely that recovery of the lower limb involves different mechanisms than recovery of hand motor function given the different roles of the feet and hands in everyday life: the former is often involved in locomotion with a strong influence of subcortical/spinal sources (Jahn et al., 2008; Yeo et al., 2011) while the hand has a special role in finely tuned, often unilateral movements (Dum & Strick, 2005; Lotze et al., 2012; Stinear et al., 2007). The differences in their behavioral roles are represented in the size of their somatotopic representations, with the hand region covering much larger parts of cortex than the foot representation (Rasmussen & Penfield, 1947). Furthermore, the cortical control of lower limb movements in healthy subjects has frequently been reported to be differentially organized compared to movements of the upper limbs. In particular, neural activity and motor network connectivity seem to be less lateralized for lower limb movements, and also interhemispheric inhibition seems to be less developed for movements of the feet compared to upper limb movements (Kapreli et al., 2006; Knaepen, Mierau, Tellez, Lefeber, & Meeusen, 2015; Luft et al., 2002; Miyai et al., 2001; Nakata, Domoto, Mizuguchi, Sakamoto, & Kanosue, 2019; Volz, Eickhoff, Pool, Fink, & Grefkes, 2015; Young et al., 2004). These differential patterns of cortical control may imply that cortical reorganization following stroke might also differ for the neural dynamics underlying upper and lower limb movements. Support for this hypothesis stems from a few functional imaging studies in stroke patients investigating disturbed lower limb function. While leg movements after stroke elicited a more bihemispheric pattern of activation, a stronger impairment of the paretic leg correlated with higher levels of neural activity in the contralesional sensorimotor cortex and the SMA (Burke, Dobkin, Noser, Enney, & Cramer, 2014; Enzinger et al., 2008; Enzinger et al., 2009; Kim et al., 2006). Further, higher levels of activity in the ipsilesional primary sensorimotor cortex indicated better foot motor performance (Burke et al., 2014; Enzinger et al., 2009; Forrester, Wheaton, & Luft, 2008). Finally, premotor areas were found to exhibit less activity changes during movements of the stroke‐affected leg compared to hand movements (Enzinger et al., 2009). Differential and heterogeneous results regarding neural activity and connectivity of lower limb movements after stroke are also found with respect to the administered task and motor function, that is, unilateral, single‐joint movement and bilateral, multi‐joint movements of the lower limbs to the point of higher functional scores and gait measurements, which likewise affected cortical brain activation as well as anatomical and functional connectivity (Peters et al., 2018; Promjunyakul, Schmit, & Schindler‐Ivens, 2015; Vinehout, Schmit, & Schindler‐Ivens, 2019). These differential patterns lead to the question whether differential adaptations of the motor network dynamics of the upper versus the lower limb drive functional reorganization. Such differences might help to explain the clinical observation that the recovery of lower limb function is typically faster and often better compared to the recovery of hand motor function (Desrosiers et al., 2003; Twitchell, 1951).

To address this question, we used fMRI and a sparse‐sampling acquisition protocol to investigate neural activity underlying movements of the paretic hand and foot in 12 left‐hemispheric stroke patients and 12 healthy control subjects. All patients suffered from persisting mild to moderate deficits in their chronic poststroke phase. Dynamic causal modeling (DCM, Friston, Harrison, & Penny, 2003) was used to determine the effective connectivity within a bilateral cortical network comprising core regions of the motor system engaged in isolated movements of the upper and lower limbs: M1_hand_ and M1_foot_ as the limb‐specific representations within the primary motor cortex as well as SMA and PMv as premotor regions.

This experimental setup enabled us to directly compare not only the neural activation underlying movements of the upper and lower limbs but also the respective network perturbations not only across different studies and tasks but in the same paradigm. On these grounds, we focused on distinct cortical regions in ipsi‐ as well as contralesional hemispheres and simple comparable movements of the affected hand and foot. As we investigated well‐recovered stroke patients, we expected to obtain a very much restored neural activation pattern that should be more lateralized in case of upper limb compared to lower limb movements. Furthermore, we hypothesized that possible involvement of the contralesional hemisphere would increase with greater persistent motor impairment. Whereas cortical reorganization of upper and lower limb movements after stroke would share these principles, we also expected limb‐specific differences underlying recovery of function based on our findings reported in Volz, Eickhoff, et al. (2015) in healthy subjects. Accordingly, we assumed a stronger supporting influence of the contralesional hemisphere such as a positive interhemispheric coupling between the cortical representation of the feet compared to a more lateralized pattern of excitatory and inhibitory couplings during upper limb movements.

## 2 MATERIALS AND METHODS

### 2.1 Participants

We recruited 12 chronic (i.e., mean 17 months, range 5–28 months) ischemic stroke patients (9 male, mean age 70.4 years, ±8.9 *SD*, range 58–85 years). Data were acquired in April 2014. All patients had initially been admitted to the Stroke Unit of the Neurological Department of the University Hospital Cologne because of an acute, first‐ever ischemic stroke causing right‐sided hemiparesis affecting both the right hand and right foot. Dependent on the lesion site, paresis can be more prominent on the upper or the lower limbs. As the middle cerebral artery territory is most frequently lesioned by ischemic stroke (Bogousslavsky, van Melle, & Regli, 1988; Olsen, Skriver, & Herning, 1985; Treadwell & Thanvi, 2010), the right hand was slightly more affected than their right foot in 7 of our 12 stroke patients. Lesions were located cortically (*n* = 3), subcortically (*n* = 5), or both (*n* = 4) (Table 1). The lesion overlap was found at the course of the corticospinal tract with highest overlap at the level of the posterior limb of the internal capsule (Supplementary Figure S2). Note that none of the lesions covered the regions of interest (ROIs) used for DCM (Yousry et al., 1997). Patients were not included in case of severe neuropsychological deficits like aphasia, neglect, or dementia. Furthermore, no contraindications for MRI were allowed. Severe leukaraiosis as determined by MRI was another exclusion criterion as this might affect the BOLD signal. The neurological deficit of the patient was rated using the National Institute of Health Stroke Scale (NIHSS, Brott et al., 1989). This standard clinical scale describes the neurological status after stroke based upon functions such as consciousness, visual fields, sensation, movement, speech, and language (range 0–42; 0 = no deficit, 42 = most severe deficits; http://www.ninds.nih.gov/doctors/NIH_Stroke_Scale.pdf).

**TABLE 1:** Patient characteristics

Patient	Gender	Age	Lesional hemisphere	Lesion site	Lesion volume (ml)	Time poststroke (months)	NIHSS (acute_phase)	NIHSS (day of experiment)	MI—upper limb	MI—lower limb
									(day of experiment)	
P01	M	70	L	Precentral gyrus, SPL	2.68	10	1	0	99	99
P02	F	75	L	Precentral/postcentral gyrus, IG, ext. capsule	22.39	28	7	2	78	75
P03	M	61	L	Pons	3.29	28	10	0	99	99
P04	M	73	L	Int. capsule, corona radiata, BG, IG, STG	11.90	25	19	0	76	75
P05	M	66	L	Internal capsule (crus posterius)	2.05	12	12	1	42	50
P06	M	59	L	Int. capsule, corona radiata, BG, IG, ITG	12.43	5	15	0	99	99
P07	M	58	L	Internal capsule (crus posterius)	1.54	5	10	0	99	99
P08	M	69	L	Caudate, corona radiata, TL, IG	64.60	21	7	1	99	99
P09	M	67	L	Pons	0.30	11	3	0	99	99
P10	F	83	L	Corona radiata, IG	2.39	10	6	0	99	99
P11	F	79	L	Caudate, corona radiata, Int. capsule	6.63	21	11	0	91	99
P12	M	85	L	Ventral precentral gyrus	2.41	28	5	0	99	91
Mean (*SD*)		70.4 (8.9)			11.84 (18.72)	17 (9.1)	8.8 (5.1)	0.3 (0.7)	89.9 (17.3)	90.3 (15.7)

*Note:* NIHSS (0–42, higher scores reflect greater neurological deficit). MI (0–99, higher scores reflect better recovery).

Abbreviations: BG, basal ganglia; ext., external; F, female; IG, insular gyrus; int., internal; ITG, inferior temporal gyrus; L, Left; M, male; MI, Motricity Index; NIHSS, National Institute of Health Stroke Scale; SPL, superior parietal lobule; STG, superior temporal gyrus; TL, temporal lobe.

We also assessed the Motricity Index (MI, Demeurisse, Demol, & Robaye, 1980) on the day of the fMRI experiment. The MI is a brief motor rating scale based on movements of the proximal, middle, and distal joints of arms and legs, which are classified according to whether they can be performed against gravity or even against resistance. Subjects had an average MI of the affected upper and lower limb of 180.2 (range 92–198, possible maximum score: 198) at the day of the experiment. Table 1 summarizes the relevant patient characteristics.

Twelve healthy volunteers without any history of neurological, psychiatric or relevant orthopedic disease served as an age‐matched control group (9 males, mean age 65.1 years, ±10.5 *SD*, range 52–83 years; age difference between groups: *t*(22) = 1.34, *p* = .195). According to the Edinburgh Handedness Inventory (Oldfield, 1971), which also comprises an item addressing footedness (“Which foot do you prefer to kick with?”), all subjects except one patient were right‐handed. To further test whether this left‐handed subject introduced any bias in the brain imaging data, analyses were re‐computed after removing this subject (see Section 3). All subjects participated after giving written informed consent. The study was performed following the declaration of Helsinki and had been approved by the local ethics committee.

### 2.2 Motor behavior

Motor impairment was quantified using the (1) maximum finger tapping frequency, (2) maximum foot tapping frequency, (3) maximum grip force, and (4) maximum contraction force of the ventral flexion of the foot. All tests were performed separately with both hands and feet. Finger tapping should be performed on a button‐device as fast as possible with the index finger (out of the base joint) while subjects were instructed to maintain a stable position with their wrist on the table to prevent movements of the forearm. During the foot tapping condition subjects were seated on a chair to maintain constant angles of the hip and knee joints of about 90° and were required to tap with their foot on the floor as fast as possible (out of the ankle). Meanwhile their heel should maintain a stable position on the floor. A cube (size 7.5 cm) was placed in front of the foot and should be reached in height during every foot tap in order to standardize tapping movements. Tapping frequency was assessed as average performance across five trials (3 s each), which were separated by 30 s rest to prevent fatigue.

The maximum force of the hand and foot were measured using a vigorimeter ball (Martin, Tuttlingen, Germany). For maximum grip force assessment, subjects were instructed to squeeze the ball with a whole‐hand grip as hard as possible in three trials separated by 30 s rest. This kind of task engaged especially the long and short finger flexors. For maximum foot flexion force assessment, the vigorimeter ball was placed underneath the forefoot and subjects were instructed to push the ball into the ground (out of the ankle) as hard as possible. The sitting position of the participants was carefully observed and corrected to ensure a stable posture with both hips and knees in about 90° flexion and the heel fixed on the floor to prevent force transduction from the proximal hip or upper leg. Hence, this kind of task especially engaged the plantar flexors. Similar to the finger tapping task, the maximum force of the hand (grip strength) and foot (plantar flexion) was assessed as average performance across three trials (3 s each), which were separated by 30 s rest to prevent fatigue.

### 2.3 Experimental design

**FIGURE 1:**
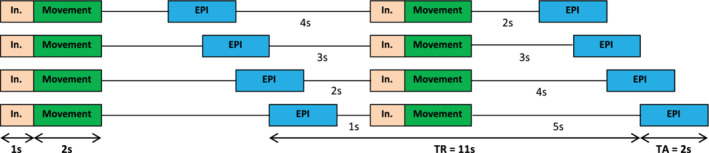
Sparse sampling design: Movement execution and image acquisition (echo planar imaging [EPI]) are performed separately using a variable time interval in between, thus allowing a sampling of the hemodynamic response independent of movement execution, thereby avoiding movement artifacts induced by task‐related head motion. In., instruction; TA, time of acquisition; TR, time of repetition

The experimental paradigm was adopted from an fMRI experiment on hand and foot movements in healthy young subjects published by our group (Volz, Eickhoff, et al., 2015). Like in our previous study, we applied an event‐related “sparse sampling” design (Figure 1) to reduce the impact of movement‐associated head motion artifacts in the fMRI time series. This is important when comparing fMRI signals resulting from the upper and lower limbs because especially leg movements tend to translate into spine and head displacement, increasing head motion artifacts (Seto et al., 2001; Weiss et al., 2013). Sparse sampling minimizes head movement artifacts by decoupling movement execution from image acquisition (Dresel et al., 2005; Volz, Eickhoff, et al., 2015). Images are acquired after movement execution, that is, during the rest period following the movement, which still contains evoked hemodynamic responses due to the time lag of the neural activity and its hemodynamic response (approx. 3–5 s until maximum response), as described by the hemodynamic response function (HRF, canonical HRF as used in SPM). Accordingly, images were acquired 2–5 s (temporal jitter) after a block of movements, leaving enough time for residual movements of the body to settle, and allowing the participants to lie as still as possible during the actual image acquisition (Amaro et al., 2002; Dresel et al., 2005). By varying the time between movements and image acquisition, we sampled the movement‐induced hemodynamic response at different time points, accounting for regional differences in HRF peaks. A disadvantage of this method, compared to a classical block design, lies in its decreased statistical power due to the lower number of images per condition. However, simple motor tasks as used here typically result in highly robust BOLD‐signal changes compared to more complex, for example, cognitive tasks and are therefore especially suited for sparse sampling designs (Volz, Eickhoff, et al., 2015).

In the present study, subjects performed visually cued movements with their (left or right) hand or (left or right) foot in separate blocks. An instruction was displayed on a shielded thin‐film transistor screen at the rear end of the scanner (visible for the subject via a mirror mounted to the MR head coil) for 1 s and indicated which limb to move in the upcoming trial. Movements (the fist closure or the foot flexion) were cued by a red blinking circle at a rate of 1.5 Hz for 2 s, resulting in three movements per block. Each block (including instruction, movement execution, jitter, and echo planar imaging [EPI] acquisition) took 11 s and was repeated 20 times. Additionally, 20 “null events” (black screen), during which subjects were instructed to rest and lie still, served as a resting baseline. The whole experiment comprised 100 trials (movement conditions and null events), presented in a randomized order across subjects, and lasted about 18 min. Our pilot experiments showed that this duration yields sufficient fMRI signal for DCM modeling, and did not lead to significant fatigue, which is important when scanning stroke patients with motor deficits.

All subjects were familiarized with the task both outside and inside the scanner before the fMRI experiment started. Motor performance regarding the moved limb and the number of movements were documented by an experimenter standing next to the scanner.

### 2.4 Image acquisition

Functional MR images were recorded on a Siemens Trio 3.0 T scanner (Siemens Medical Solutions, Erlangen, Germany) using a gradient EPI sequence with the following parameters: time of repetition (TR) = 11 s, time of acquisition (TA) = 2 s, time of echo (TE) = 30 ms, field of view (FoV) = 220 mm, flip angle = 90 °, voxel size = 3.4 × 3.4 × 3.4 mm^3^, slices = 30, distance factor = 33% (1.1 mm interslice‐gap), volumes = 105. Image slices covered both hemispheres and the brain stem to lower parts of the pons and cerebellum. This voxel size has been shown to allow for a robust estimation of DCM connectivity in the motor system (Pool, Rehme, Fink, & Eickhoff, 2013; Pool et al., 2018). As explained in detail above, a sparse sampling design was used (Figure 1): The EPI volume (depicted in blue) was recorded every 11 s for the duration of 2 s. After movement execution in the scanner (depicted in green), a variable delay of 2–5 s was used before fMRI data collection (EPI) to allow a sampling of the hemodynamic response (peaking around 3–4 s) independent of movement execution (Volz, Eickhoff, et al., 2015). In addition to the fMRI volumes, high‐resolution T1‐weighted structural images were scanned (TR = 2,250 ms, TE = 3.93 ms, FoV = 256 mm, voxel size = 1.0 mm^3^, slices = 176), which served as anatomical reference.

### 2.5 Image processing

#### 2.5.1 Preprocessing and general linear model

All neuroimaging analyses were performed using Statistical Parametric Mapping (SPM8, Wellcome Department of Imaging Neuroscience, London, UK, http://www.fil.ion.ucl.ac.uk, release 2013). We used SPM8 to ensure comparability with our previous DCM studies investigating the motor system of both healthy subjects and stroke patients (e.g., Grefkes, Nowak, et al., 2008; Pool et al., 2013; Rehme, Eickhoff, et al., 2011; Volz, Eickhoff, et al., 2015). After realignment of the EPI volumes and coregistration with the anatomical T1 image, all volumes were spatially normalized to the standard template of the Montreal Neurological Institute (MNI, Canada) employing the unified segmentation approach (Ashburner & Friston, 2005). For spatial normalization, a binary lesion mask was used. Finally, an isotropic smoothing kernel of 8 mm full width at half maximum was applied.

For statistical analysis, boxcar vectors for each condition (i.e., movement of the right hand, left hand, right foot, and left foot) were convolved with a canonical HRF to create regressors of interest for the general linear model (GLM). The time series in each voxel were high‐pass filtered at 1/128 Hz to remove low‐frequency drifts. Movement parameters as assessed by the realignment algorithm were treated as covariates on the single subject level to exclude movement‐related variance from the image time series. All subjects did not move more than 2 mm in *x*, *y*, and *z* directions. In addition, activation patterns were checked in each individual subject, which was a necessary prerequisite for DCM for which VOIs are determined at the single subject level. The parameter estimates obtained from the GLM at the single subject level (*n* = 24) were entered into t‐statistics for linear contrasts comparing “moving limb (e.g., right hand) vs. rest” in each subject group (i.e., patients and controls) as well as “patients versus controls” for each limb (e.g., patients > controls when moving the right hand). Voxels were considered significant when passing a statistical threshold of *p* < .05, family‐wise error (FWE)‐corrected at the cluster level (cluster‐forming threshold *p* < .001).

#### 2.5.2 Interaction contrast and correlations

To identify limb‐specific differences in the reorganization patterns, we computed an analysis of variance (ANOVA) to test for possible interaction effects of “group” (i.e., patients vs. controls) and “limb” (i.e., the affected right hand vs. affected right foot). Furthermore, we performed correlations with and without the factors “age,” “sex,” and “time since stroke” as covariates on the contrast estimates in each ROI to test for correlations between the neural activity during movements of the affected limb and behavioral scores (*p* < .05, false discovery rate (FDR)‐corrected for multiple comparisons, two‐tailed). Subjects with values between 1.5 and 3 times the interquartile range were considered to represent statistical outliers (4.5% of data) and therefore excluded from the respective correlation analysis to ensure that they did not drive the results.

#### 2.5.3 ROIs analysis

In order to increase statistical sensitivity, we defined 10 ROIs representing core regions of the motor system in both hemispheres (Grefkes, Eickhoff, et al., 2008; Kapreli et al., 2006; Luft et al., 2002; Volz, Eickhoff, et al., 2015; Wang et al., 2011): As in previous studies, the ROIs were defined as spheres (radius: 4 mm) and centered on the representations of the hand (M1_hand_) and foot (M1_foot_) within the primary motor cortex, the SMA, the PMv, and the PMd (Meier et al., 2018; Pool et al., 2018; Volz, Eickhoff, et al., 2015).

Coordinates were selected by identifying activation maxima in the BOLD data within predefined anatomical constraints: M1_hand_ on the rostral wall of the central sulcus at the “hand knob” formation (Diekhoff et al., 2011; Yousry et al., 1997), M1_foot_ at the paracentral lobule (Lotze et al., 2000), SMA on the mesial wall within the interhemispheric fissure between the paracentral lobule (posterior landmark) and the anterior commissure (Picard & Strick, 2001), PMv in the precentral sulcus close to the inferior precentral gyrus and pars opercularis (Rizzolatti, Fogassi, & Gallese, 2002), and PMd at the junction of the superior frontal sulcus and the superior part of the precentral sulcus (Tomassini et al., 2007).

As simple unilateral limb movements typically result in mainly contralateral activation of the (primary) motor cortex, the respective M1 regions were identified using the contrast “movement of the contralateral limb versus rest” (e.g., “right hand vs. rest” for identification of the left [ipsilesional] M1_hand_). Premotor ROIs were specified using a conjunction analysis across both movements of the upper and lower limb (e.g., “right hand vs. rest” ∩ “right foot vs. rest” for left SMA, PMv, and PMd). Voxels in the ROIs surviving a FWE‐small‐volume‐corrected threshold of *p* < .05 were considered significant. The group coordinates of the healthy control group served as functional localizers to center the ROIs for the small volume correction procedure (search diameter: 8 mm, see Table 2).

**TABLE 2:** MRI activation maxima (group contrast healthy controls) used as regions of interest

Brain region	MNI coordinates			*p*	*T*	*Z*
	*x*	*y*	*z*			
M1FL	−4	−30	67	<.001	6.69	6.00
M1FR	6	−30	72	<.001	8.00	6.92
M1HL	−34	−26	53	<.001	8.77	7.41
M1HR	32	−23	64	<.001	7.06	6.27
PMvL	−53	1	40	<.001	4.36	4.14
PMvR	52	8	37	.001	3.84	3.68
PMdL	−33	−6	60	<.001	5.30	4.93
PMdR	34	−11	52	*No suprathreshold clusters*		
SMAL	−4	−10	66	<.001	5.62	5.18
SMAR	4	−5	65	<.001	5.23	4.87

Abbreviations: L, left; M1F, primary motor cortex (M1) of the foot; M1H, M1 hand; MNI, Montreal Neurological Institute; MRI, magnetic resonance imaging; PMd, dorsal premotor cortex; PMv, ventral premotor cortex; R, right; SMA, supplementary motor area.

### 2.6 Dynamic causal modeling

DCM (Friston et al., 2003) was used to estimate effective connectivity between the motor areas outlined above. We used DCM rather than other approaches to evaluate effective connectivity such as Granger causality mapping in order to warrant comparability with other DCM studies on motor system connectivity (Boudrias et al., 2012; Grefkes, Nowak, et al., 2008; Pool et al., 2013; Rehme, Eickhoff, et al., 2011; Volz, Eickhoff, et al., 2015). Moreover, we previously compared differential motor network dynamics underlying upper and lower limb movements using DCM in healthy subjects (Volz, Eickhoff, et al., 2015), thus representing a physiological baseline for our current assessment of stroke patients. Furthermore, GC mappings, based on the concept of temporal precedence, might be problematic in case of substantial inter‐regional variability of the hemodynamic response (David et al., 2008) as, for example, in stroke patients (Grefkes & Fink, 2011). DCM represents a computational framework which considers the brain as a dynamic system in which external perturbations (inputs) cause changes in neuronal activity or inter‐regional coupling strength (Eickhoff & Grefkes, 2011; Friston et al., 2003). DCM computes three sets of parameters for a given model: (a) the endogenous coupling independent of the experimental condition (DCM‐A matrix); (b) condition‐dependent coupling evoked by the experimental conditions, that is, movement of the left and right hand and foot, respectively (DCM‐B matrix); and (c) the direct experimental input to the system that drives regional activity (DCM‐C matrix). As DCM models predict the neuronal response at any particular time point, it can account for region‐specific sampling times and, therefore, also be used for sparse imaging data (Kiebel, Klöppel, Weiskopf, & Friston, 2007; Kumar, Stephan, Warren, Friston, & Griffiths, 2007; Volz, Eickhoff, et al., 2015).

The first eigenvariate of the effects of interest adjusted time series extracted from 8 of the 10 ROIs used in the ROIs analysis served for constructing DCM. These are M1_hand_, M1_foot_, SMA, and PMv, in both hemispheres. The group coordinates of the healthy control group (given in Table 2) served as functional localizers to center the ROIs for the small volume correction procedure (search diameter: 8 mm). PMd was not included in the DCM analysis as it was not activated in the between‐subjects group contrast. The number of areas that can be included in a model is limited for computational reasons (Stephan et al., 2010). Given the lack of BOLD activity differences, PMd was considered to be less relevant compared to the other ROIs included in the DCMs. One healthy subject had to be excluded from the DCM analysis because of missing activity in the left SMA ROI even at a threshold of *p* < .1. Consequently, we excluded one subject from the patient group to keep both groups homogeneous with respect to gender and age. Thus, 22 subjects were included in the connectivity analyses. Table 3 provides the coordinates of all ROIs.

**TABLE 3:** Individual fMRI activation maxima. Brain regions used as regions of interest for connectivity analysis and MNI coordinates are given for each subject

	M1FL			M1FR			M1HL			M1HR			PMvL			PMvR			SMAL			SMAR		
Subject	*x*	*y*	*z*	*x*	*y*	*z*	*x*	*y*	*z*	*x*	*y*	*z*	*x*	*y*	*z*	*x*	*y*	*z*	*x*	*y*	*z*	*x*	*y*	*z*
C01	−6	−27	79	7	−38	74	−42	−23	63	31	−31	68	−54	9	27	55	13	41	−4	−4	74	6	−9	73
C02	−4	−19	71	6	−17	66	−33	−26	58	46	−24	51	−53	3	26	54	2	24	−10	−36	62	6	−8	57
C03	−8	−25	74	7	−19	74	−36	−23	65	42	−22	58	−52	−2	47	52	2	39	−4	−3	65	8	−11	58
C04	−7	−30	82	7	−31	77	−38	−30	50	33	−25	50	−52	4	33	53	7	40	−4	−4	58	4	−2	56
C05	−6	−33	75	11	−31	79	−35	−30	69	40	−21	58	−57	10	34	57	8	40	−6	−12	65	8	−7	69
C06	−4	−24	66	8	−25	66	−37	−22	57	39	−20	51	−49	5	31	48	6	42	−4	−4	50	5	−6	63
C07	−5	−34	78	6	−32	73	−35	−36	67	33	−22	64	−51	1	44	50	1	45	−4	−12	63	5	−21	76
C08	−4	−24	66	8	−25	66	−39	−20	58	35	−24	65	−47	6	31	48	6	42	−9	−21	60	5	−6	63
C09	−11	−34	79	4	−34	77	−29	−35	71	31	−21	68	−52	5	19	47	9	19	−6	−27	61	5	−8	56
C10	−7	−23	76	4	−26	75	−37	−16	65	36	−28	62	−53	6	38	45	7	37	−4	−5	52	4	−7	64
C11	−7	−29	75	6	−30	77	−29	−24	70	33	−23	65	−61	18	21	61	10	16	−4	1	57	4	0	59
C12	−8	−28	75	8	−13	74	−39	−29	59	35	−27	62	−64	−11	21	61	−1	35	^a^			5	−8	61
P01	−6	−28	76	5	−42	68	−36	−28	62	34	−29	67	−52	9	29	54	15	29	−4	−4	53	4	5	50
P02^a^	−4	−29	77	9	−33	74	−41	−26	58	40	−26	59	−60	11	25	55	5	40	−4	−9	56	4	−7	59
P03	−5	−32	76	12	−34	68	−43	−33	60	43	−26	58	−52	−1	38	52	−2	41	−7	−14	55	4	−3	57
P04	−4	−32	68	8	−37	67	−34	−30	65	34	−29	59	−51	4	36	52	4	37	−6	−8	68	4	−12	64
P05	−4	−36	70	11	−26	76	−34	−27	63	34	−25	64	−48	3	38	55	0	41	−4	−7	52	5	−8	56
P06	−4	−25	72	4	−27	72	−40	−29	63	39	−20	60	−53	−2	40	52	7	44	−7	−9	75	4	−5	57
P07	−6	−30	67	8	−22	72	−44	−28	57	39	−28	59	−45	1	31	43	6	30	−4	2	66	5	−3	49
P08	−4	−34	72	10	−33	70	−33	−27	56	34	−18	48	−54	2	39	55	3	35	−4	−4	56	5	−3	50
P09	−6	−20	73	8	−25	74	−37	−28	62	39	−25	59	−53	2	37	52	6	31	−4	−9	43	4	−13	65
P10	−4	−32	75	10	−27	76	−40	−24	59	40	−29	59	−52	5	32	56	−4	37	−5	−4	55	6	−6	53
P11	−5	−27	75	7	−24	71	−35	−23	68	45	−21	55	−56	−5	37	58	−6	39	−4	−3	51	4	−5	52
P12	−4	−21	72	6	−28	75	−41	−23	60	39	−19	58	−52	1	42	59	1	27	−4	−9	51	4	−3	57
Mean	−6	−28	74	8	−28	73	−37	−27	62	37	−24	59	−53	4	33	53	4	35	−5	−7	58	5	−7	59
*SD*	2	5	4	2	7	4	4	5	5	4	4	6	4	6	7	5	5	8	2	7	8	1	5	7

Abbreviations: C, healthy control; fMRI, functional magnetic resonance imaging; L, Left; M1F, primary motor cortex (M1) of the foot; M1H, M1 hand; MNI, Montreal Neurological Institute; P, patient; PMv, ventral premotor cortex; R, Right; *SD*, standard deviation; SMA, supplementary motor area.

^a^ Coordinate not determinable due to insufficient activation: C12 and P02 (matched subject) excluded from further DCM analyses.

Based on previous studies on structural connectivity in macaque monkeys we assumed endogenous connections (DCM‐A matrix) between all selected ROIs (please see Supplement Figure 1 for further details), that is, between SMA (bihemispheric) and ipsilateral and contralateral M1 (Rouiller et al., 1994), between SMA and ipsilateral as well as contralateral PMv (Boussaoud, Tanné‐Gariépy, Wannier, & Rouiller, 2005; Luppino, Matelli, Camarda, & Rizzolatti, 1993), between PMv and both ipsi‐ and contralateral M1 (Rouiller et al., 1994), as well as homotopic transcallosal connections among M1‐M1 (Rouiller et al., 1994), SMA‐SMA (McGuire, Bates, & Goldman‐Rakic, 1991), and PMv‐PMv (Boussaoud et al., 2005). As the task‐specific modulation of interregional coupling (DCM‐B matrix) may not necessarily affect all possible anatomical connections, a total of 12 alternative connectivity models representing biologically plausible hypotheses on interregional coupling were constructed (please see Section 2.7 and Supplemental Figure 1 for further explanation). We assumed the motor tasks to directly impact on the activity of all premotor regions (bilateral SMA and PMv), which were accordingly defined as input regions (DCM‐C) for all conditions in all models (Volz, Eickhoff, et al., 2015; Wang et al., 2011). Please note that DCM‐A is independent of the experimental input function u as intrinsic connectivity reflects the context‐independent part of interregional coupling.

### 2.7 Bayesian model selection

Condition‐dependent coupling modulations may not affect all hypothesized endogenous connections (Penny, Stephan, Mechelli, & Friston, 2004; Stephan, Penny, Daunizeau, Moran, & Friston, 2009). Based on the DCM‐A matrix, we, therefore, set up 12 alternative models of varying complexity representing biologically plausible hypotheses on interregional coupling among ROIs during movement of the left and right hand and foot (DCM‐B matrix). Starting from a fully connected DCM‐B matrix with 56 connections we systematically reduced the number of connections regarding the presence of modulatory interhemispheric and intrahemispheric effects (for detailed schemas, please see Supplementary Figure S1). After estimation of all 12 models, we applied random effects Bayesian model selection (BMS) to identify the most likely model given the data (Stephan et al., 2009).

### 2.8 Statistical analysis

#### 2.8.1 Behavioral data analysis

Statistical analysis of motor behavior was conducted using the software SPSS Statistics (version 24, IBM SPSS Inc.). As we were specifically interested in the neural reorganization pattern of the networks controlling upper‐ compared to the lower‐limb movements, we focused the analyses on movements of the right hand and foot, thus the affected limbs in our group of left‐hemispheric stroke patients. A mixed ANOVA with the between‐subject factor “group” (two levels: patients and healthy controls, *n* = 24) and the within‐subject factor “motor behavior” (four levels: finger and foot tapping, grip and foot force) was calculated. When sphericity was violated, Greenhouse–Geisser correction was applied. In case of significant interaction effects, two‐sample two‐sided post hoc *t* tests were conducted to reveal significant differences in motor performance of the right hand and foot. *p*‐Values passing the statistical threshold of *p* < .05 were considered to be significant.

#### 2.8.2 Analysis of connectivity

Coupling parameters were tested for statistical significance using one‐sample two‐sided *t* tests (*p* < .05) and corrected for multiple comparisons (FDR‐corrected). To test for differences in endogenous connectivity (DCM‐A matrix) between stroke patients and healthy controls, we computed a mixed ANOVA including the factors “group” (two levels: patients and controls, *n* = 22) and “connections” (37 levels, connections with significant coupling parameters). If sphericity assumptions were violated, the Greenhouse–Geisser correction was applied. Post hoc *t* tests were calculated only in case of significant interaction effects. Partial eta‐squared was calculated to capture the effect size of the statistical results. Connections modulated by movements of the affected right hand or right foot were identified in the DCM‐B matrix of the “winner model” according to BMS. Again, one‐sample two‐sided *t* tests and paired two‐sided *t* tests were used to test for significant coupling parameters and group differences. As *p*‐values did not survive the FDR‐correction for multiple comparisons, results are reported at an uncorrected statistical threshold of *p* < .05 and, therefore, should be interpreted with caution (please see Sections 3 and 4 for further explanation).

To investigate the putatively behavioral relevance of the coupling parameters, we computed Pearson's correlations between coupling parameters of significant connections and the tapping frequency or grip/foot strength, respectively. The significance threshold was defined at *p* < .05, two‐tailed.

## 3 RESULTS

### 3.1 Behavioral results

When comparing the NIHSS scores assessed in the acute stroke phase and in the chronic phase on the day of the fMRI experiment, each and every patient showed recovery of function with respect to the global neurological deficit (NIHSS in the acute phase: mean 8.8 ± 5.1, range 1–19; NIHSS on the day of the fMRI experiment: mean 0.3 ± 0.7, range 0–2; *Z*_(*N* = 12)_ = −3.06, *p* = .002; please also cf. Table 1).

**FIGURE 2:**
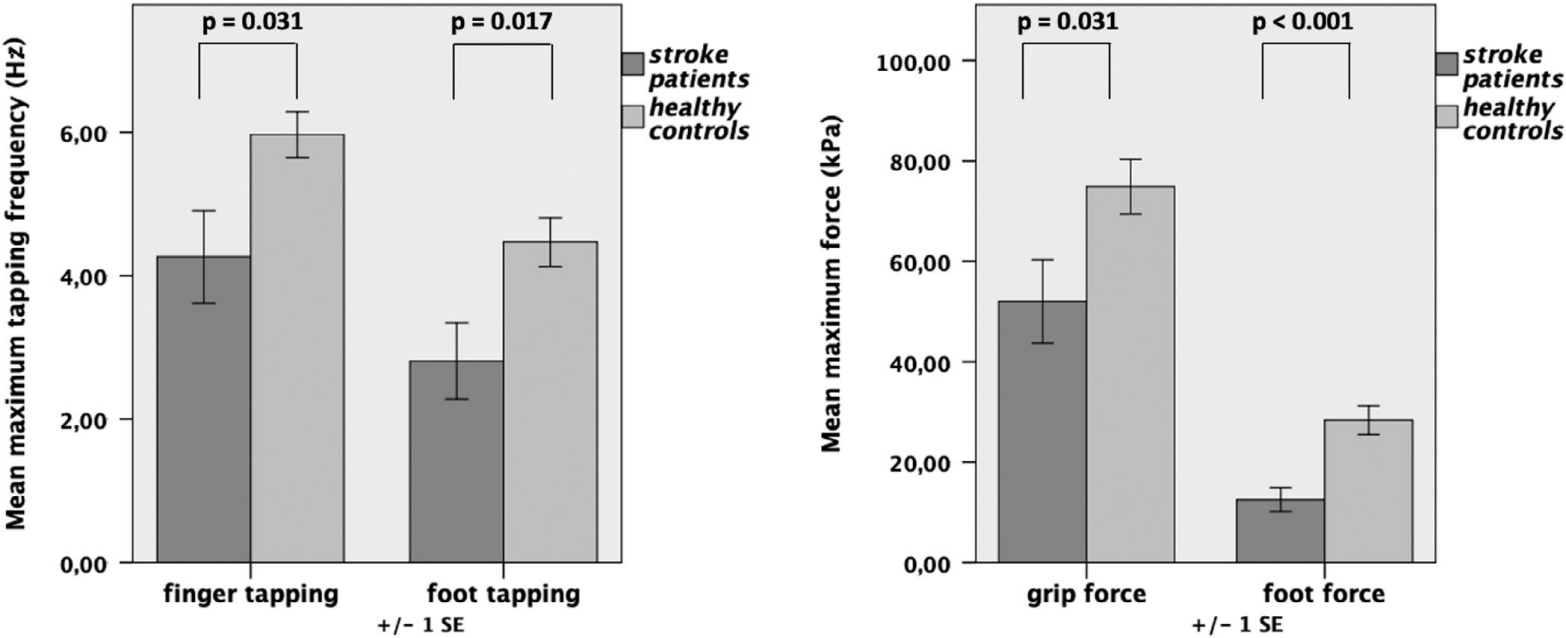
Maximum motor performance: Stroke patients (dark gray column) performed significantly worse in all motor tests with their affected (right) limb compared to healthy controls; *n* = 2 × 12. Error bars: *SEM*

When testing for differences in the motor behavior (cf. Section 2) using a mixed ANOVA on the factors “group” (patients and healthy controls) and “motor behavior” (finger and foot tapping, grip and foot force of the right limb), we found significant main effects of both “group” (*F*_(1,22)_ = 9.61, *p* = .005) and “motor behavior” (*F*_(1,21; 26,60)_ = 137.56, *p* < .001) as well as a significant interaction (*F*_(1,21;26,60)_ = 4.96, *p* = .029). Post hoc *t* tests demonstrated that stroke patients performed significantly worse with their affected right hand and foot in all tested domains of motor behavior compared to healthy controls (Figure 2).

Of note, motor performance inside the scanner was neither significantly different between the affected or unaffected hands nor between patients and healthy controls, due to the relative simplicity of the motor task. Hence, any differences in neural activity/connectivity (see below) are unlikely to be primarily driven by differences in overt motor performance.

### 3.2 BOLD activation

#### 3.2.1 Group contrasts

**FIGURE 3:**
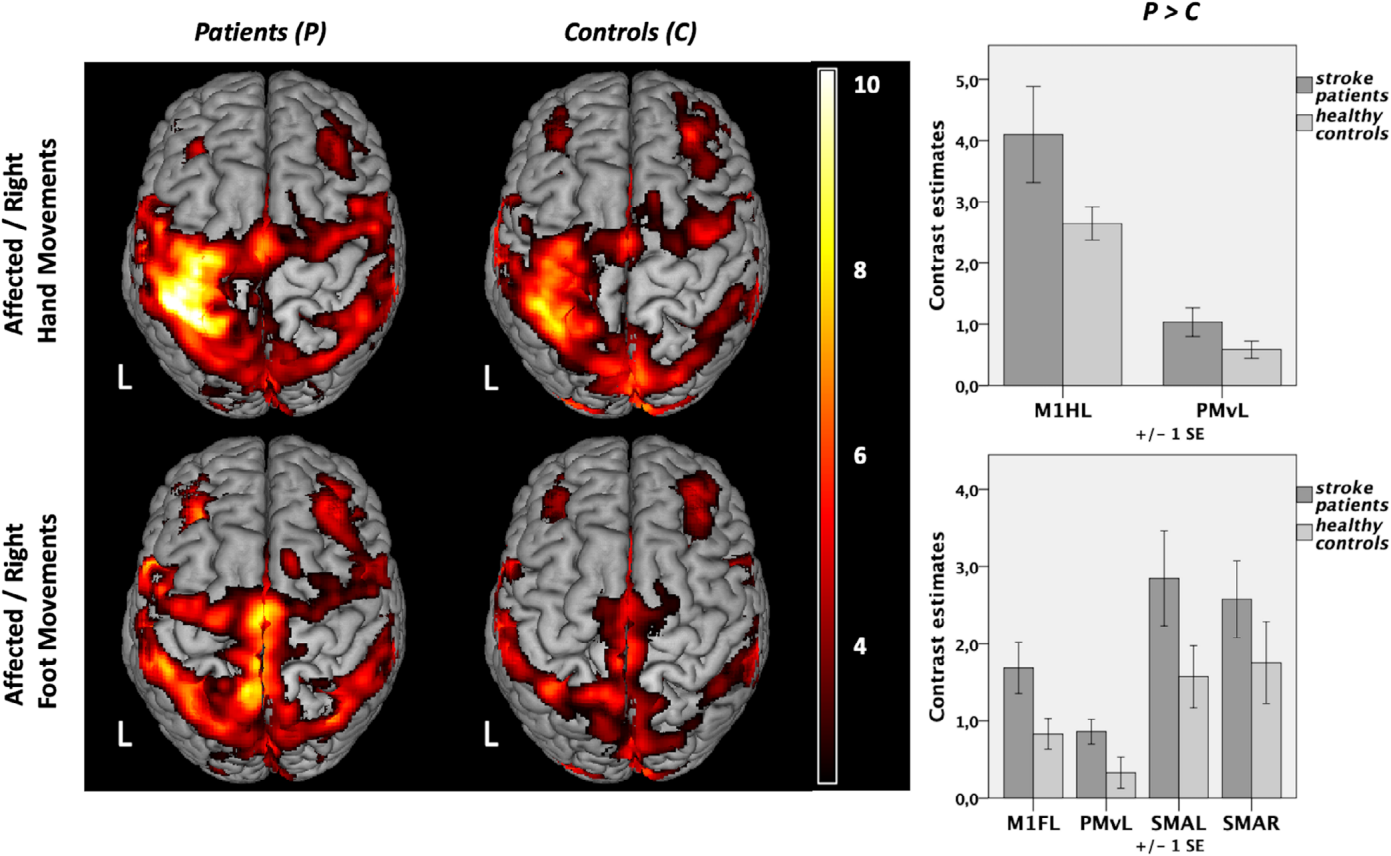
Neural activity during unilateral limb movements. Group contrasts are shown on the left (*p* < .05, family‐wise error [FWE]‐corrected at the cluster level, cluster forming threshold *p* < .001). L: left (and lesioned) hemisphere, *n* = 2 × 12. Significant group differences in regions of interest (ROIs) are depicted on the right (*p* < .05 FWE‐small‐volume‐corrected). Contrast estimates during movements of the right hand at left M1 (hand) [−35 –29 55] and left ventral premotor cortex (PMv) [−54 –1 37]; contrast estimates during movements of the right foot at left M1 (foot) [−8 –30 67], left PMv [−53 1 36], left supplementary motor area (SMA) [−5 –10 67], and right SMA [1 –7 66]

Contrasting “right hand movements” versus “rest” revealed enhanced BOLD activity within a left‐lateralized network for both stroke patients and healthy controls (Figure 3, upper left): In both groups, significant neural activity was found in contralateral sensorimotor cortex including left M1_hand_ and adjacent somatosensory cortex (S1), bilateral premotor cortex (PMv, PMd, SMA), secondary somatosensory cortex (S2), dorsolateral prefrontal and parietal cortex, as well as striate and extrastriate areas, the latter representing the processing of the visual cues. At the subcortical level, the basal ganglia including the thalamus, putamen, and pallidum as well as the cerebellum were activated (*p* < .05, FWE‐corrected at the cluster level). Testing for significant group differences, we found stronger BOLD activity in left (ipsilesional) M1 and PMv (p < .05, FWE‐small‐volume‐corrected; Figure 3, upper right) during hand movements for patients compared to healthy controls. The reverse contrast did not yield significant results, that is, controls did not show higher movement‐related activity compared to patients.

In healthy subjects, contrasting “right foot movements” versus “rest” showed a more bilateral activation pattern at the level of the sensorimotor cortex along the interhemispheric fissure compared to hand movements (Figure 3, lower left). Testing for group differences revealed that movements of the right stroke‐affected foot yielded significantly higher levels of activity in the left (ipsilesional) M1_foot_, the left PMv, as well as in bilateral SMA (Figure 3, lower right). Healthy controls did not elicit higher levels of neural activation compared to stroke patients in any ROI. Likewise, movements of the left unaffected hand and foot were associated with a lateralized activation pattern to the right hemisphere that did not differ between patients and healthy controls.

#### 3.2.2 Limb‐specific reorganization after stroke

**FIGURE 4:**
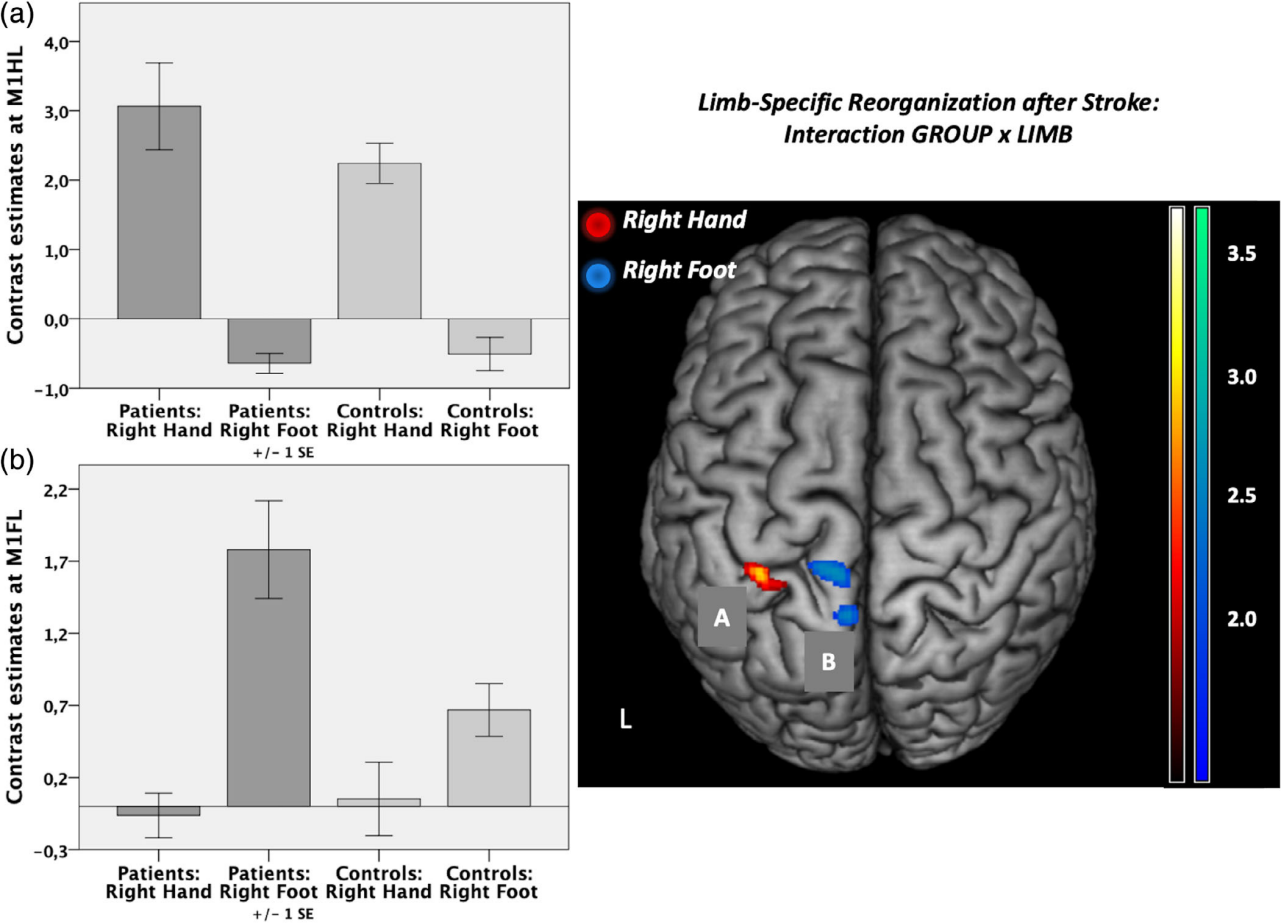
Regions of interest (ROIs) analysis. Interaction contrast “group” (patients vs. controls) and “limb” (right hand vs. right foot) as well as contrast estimates are shown. (a) Response at left M1 hand [−34 –30 53]; *p* < .05 family‐wise error (FWE)‐small‐volume‐corrected. (b) Response at left M1 foot [−8 –30 67]; *p* < .05, FWE‐corrected. L: left (and lesioned) hemisphere; *n* = 2 × 12. ROIs depicted in red reflect neural activity higher in patients compared to healthy controls for movements of the affected hand compared to the foot, whereas ROIs depicted in blue stand for neural activity higher in patients compared to controls and higher for movements of the affected foot compared to the hand

We next tested for an interaction effect of the factors “group” and “limb” in the ROIs mentioned above. That is, we sought to identify regions where stroke‐induced increases in neural activity (compared to healthy controls) were significantly higher for movements of the affected hand compared to movements of the affected foot and vice versa. This analysis identified neural activity in the left M1_hand_ to be significantly higher in patients compared to controls when patients moved their affected hand compared to moving their affected foot (Figure 4a). For the reverse contrast, that is, higher activity in patients when the foot was moved compared to hand movements, we observed significantly stronger activity in left M1_foot_ and adjacent anterior motor cortex (Figure 4b). When removing the left‐handed patient, results changed to *p* = .051 for left M1_foot_ (left M1_hand_ still *p* < .05). All other results (fMRI, DCM) remained stable when removing the left‐handed patient.

#### 3.2.3 Correlation between behavioral performance and BOLD activity

**FIGURE 5:**
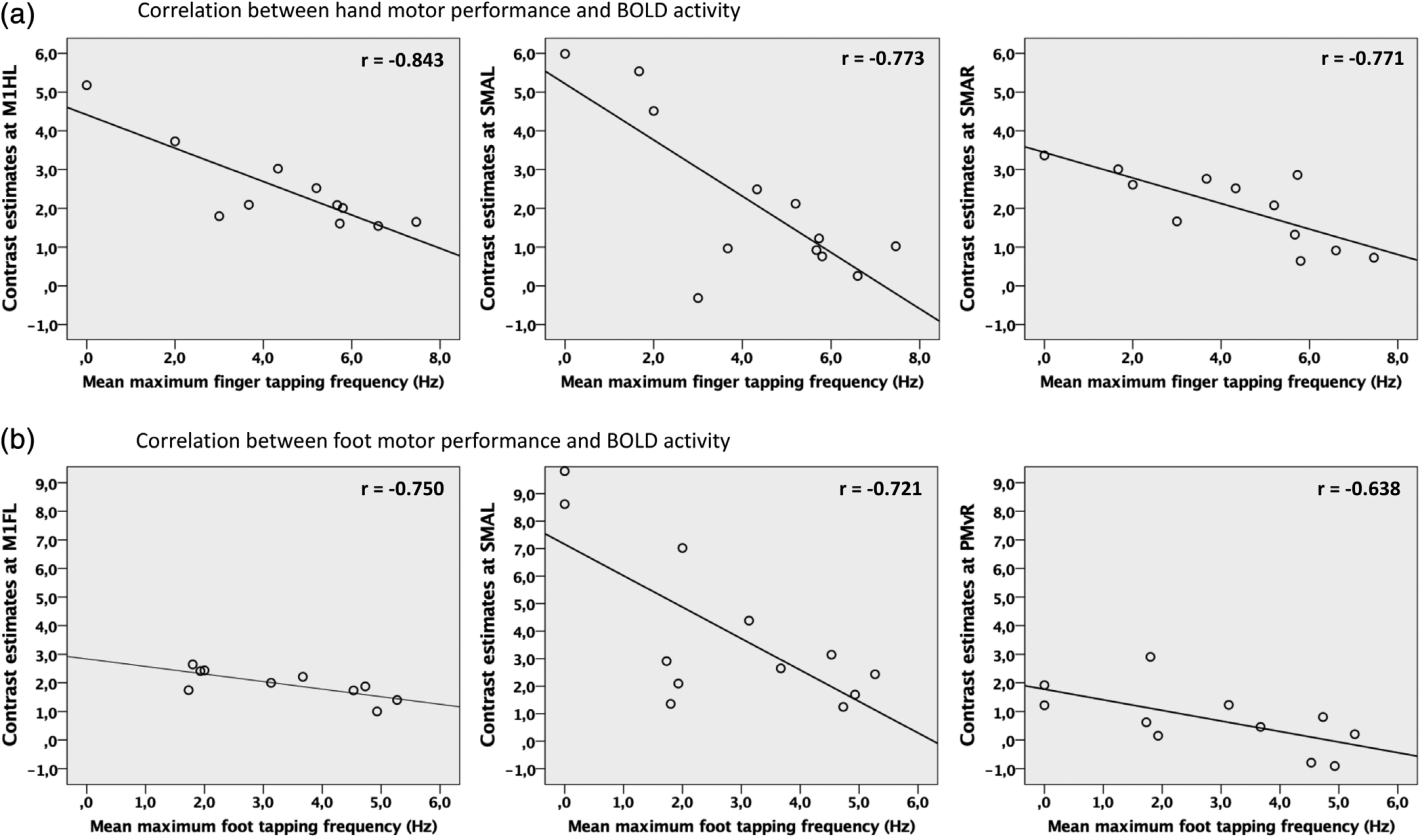
Enhanced neural activity in regions of interest (ROIs) (*p* < .05 family‐wise error (FWE)‐small‐volume‐corrected) was correlated with lower tapping frequencies of the affected limb in patients. (a) Significant correlations for movements of the affected right hand and (b) foot (*p* < .05, false discovery rate (FDR)‐corrected for multiple comparisons)

Next, we tested whether BOLD activity levels observed during movements of the hands and feet correlated with the individual performance levels assessed in the tapping tasks outside the scanner. Tapping frequencies were negatively correlated with BOLD activity for both movements of the affected right hand and affected right foot. Higher levels of activity were associated with greater motor impairment (lower tapping frequency). For hand movements, strong negative correlations were found for activity peaks in bilateral premotor ROIs comprising the ipsilesional SMA (*r* = −.773, *p* = .003, 95% CI −0.985 to 0.039) as well as the contralesional SMA (*r* = −.771, *p* = .003, 95% CI −0.944 to −0.430). In addition, we found a very strong negative correlation for activity in left M1 (hand knob) (*r* = −.843, *p* = .001, 95% CI −0.986 to −0.239) (Figure 5a). Strong negative correlations between foot tapping performance and BOLD activity were found in both hemispheres in left M1_foot_ (*r* = −.750, *p* = .012, 95% CI −0.963 to −0.338), left SMA (*r* = −.721, *p* = .008, 95% CI −0.953 to −0.123) as well as moderately strong in right PMv (*r* = −.638, *p* = .035, 95% CI −0.863 to −0.302) (Figure 5b). None of the correlations were driven by outliers and remained significant when corrected for the factors “age,” “sex,” and “time since stroke” (compare Section 2).

No significant correlations were found concerning grip force or foot force.

### 3.3 Connectivity analysis

#### 3.3.1 Bayesian model selection

**FIGURE 6:**
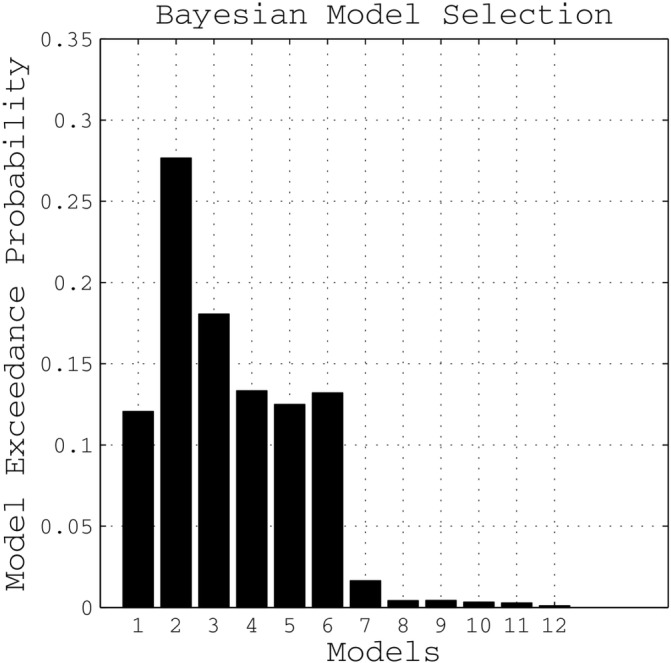
Bayesian model selection: Exceedance probability of all tested models for the entire group, *n* = 2 × 11

According to the random effects BMS, Model 2 showed the highest exceedance probability of all tested models for the entire group, as well as when separately assessing patients or controls (Figure 6). This model assumed connectivity among nearly all regions except an interhemispheric connection between PMv and SMA. On average, this “winning” model explained 39.18 ± 20.13% *SD* of the total mean variance, indicating a good fit of predicted and observed responses. It was therefore considered the most likely generative model given the data, and coupling parameters estimated for this model were used for all subsequent analyses.

#### 3.3.2 Endogenous connectivity

Endogenous connectivity (DCM‐A matrix) reflects the coupling strength from one area over another independent of the experimental condition, that is, irrespective of which limb was moved.

**FIGURE 7:**
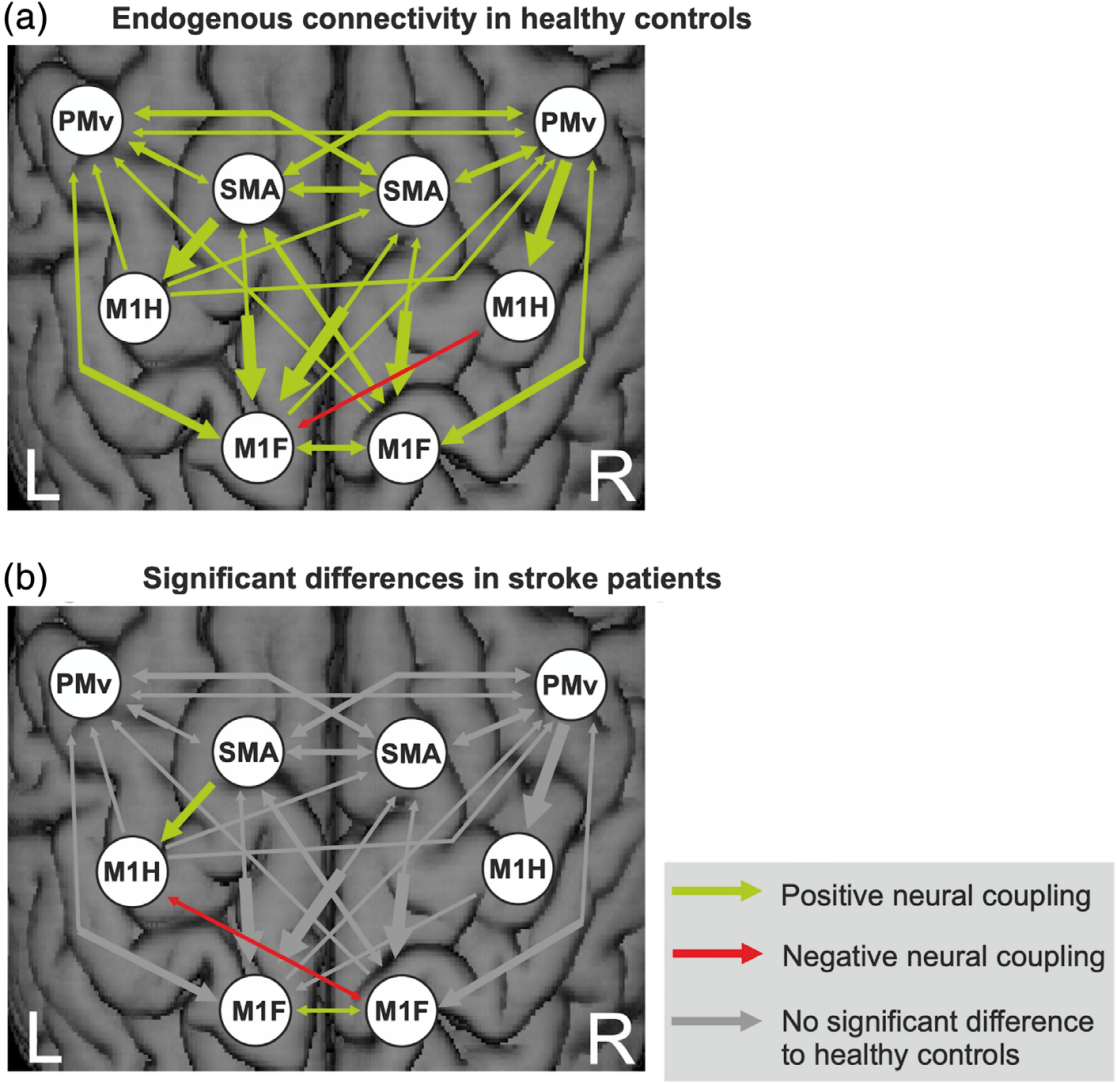
(a) Endogenous connectivity (dynamic causal modeling [DCM]‐A matrix) in healthy controls (*p* < .05, false discovery rate [FDR]‐corrected). (b) Differences compared to stroke patients (post hoc *t* test, *p* < .05). The width of each arrow corresponds to the coupling strength; *n* = 2 × 11

In healthy subjects, the DCM‐A matrix revealed a mostly symmetric network of mainly facilitating connectivity estimates (Figure 7a), that is, the connected ROIs exert positive influences among each other representing excitatory connections in the brain. Positive coupling parameters are depicted in green, whereas negative coupling parameters (red arrows) can be interpreted as inhibition of neural activity. Coupling parameters are quantified in Tables S1 and S2 of the Supplement. Premotor areas (PMv and SMA) exerted an intrahemispheric and interhemispheric facilitating influence onto each other and especially onto M1 (hand and foot). The only inhibitory influence (red arrow) was found for the interhemispheric connection originating from right M1_hand_ onto left M1_foot_. The interhemispheric (inhibitory) connection between left and right M1_hand_ did not reach statistical significance. In contrast, the interhemispheric coupling between left and right M1_foot_ was positive (Figure 7a).

When testing for significant differences in endogenous connectivity between healthy controls and left‐hemispheric chronic stroke patients, a mixed ANOVA revealed a significant main effect for “connection” (*F*_(36,720)_ = 10.33, *p* < .001, *ƞ* ^2^ = 0.341) as well as a significant interaction effect “group × connection” (*F*_(36,720)_ = 1.62, *p* = .013, *ƞ* ^2^ = 0.075), representing moderate to strong effects. Post hoc *t* tests demonstrated the connection from left SMA onto left M1_hand_ as well as the interhemispheric coupling between left and right M1_foot_ to be significantly weaker in the patients relative to the healthy controls (Figure 7b).

Of note, the coupling strength between the left SMA and the left M1_hand_ positively correlated with the mean maximum finger tapping frequency (*r* = .456, *p* = .33, 95% CI 0.041–0.708) and mean maximum grip force (*r* = .485, *p* = .22, 95% CI 0.071–0.815) in the group of 22 subjects. Hence, participants with lower motor performance featured reduced excitatory input from left SMA on left M1_hand_. However, the correlation did not reach significance in the patient group (*N* = 12) alone, reflecting that correlations should be considered cautiously in the case of small sample sizes. Nevertheless, we consider it to be a valid finding as the very same connection was found to be altered in many other stroke fMRI studies with totally independent samples, (Bajaj, Butler, Drake, & Dhamala, 2015a, 2015b; Bajaj et al., 2016; Grefkes, Nowak, et al., 2008; Sharma, Baron, & Rowe, 2009; Wang et al., 2016). Interestingly, the coupling strength between the left SMA and the left M1_foot_ was not correlated with motor behavior (foot tapping frequency and foot force). Additionally, we found a significant inhibition between ipsilesional M1_hand_ and contralesional M1_foot_ in the patient group but not in the healthy controls. However, unlike the SMA‐M1 connection, there was no correlation with behavior for the M1‐M1 connection.

#### 3.3.3 Condition‐dependent connectivity

**FIGURE 8:**
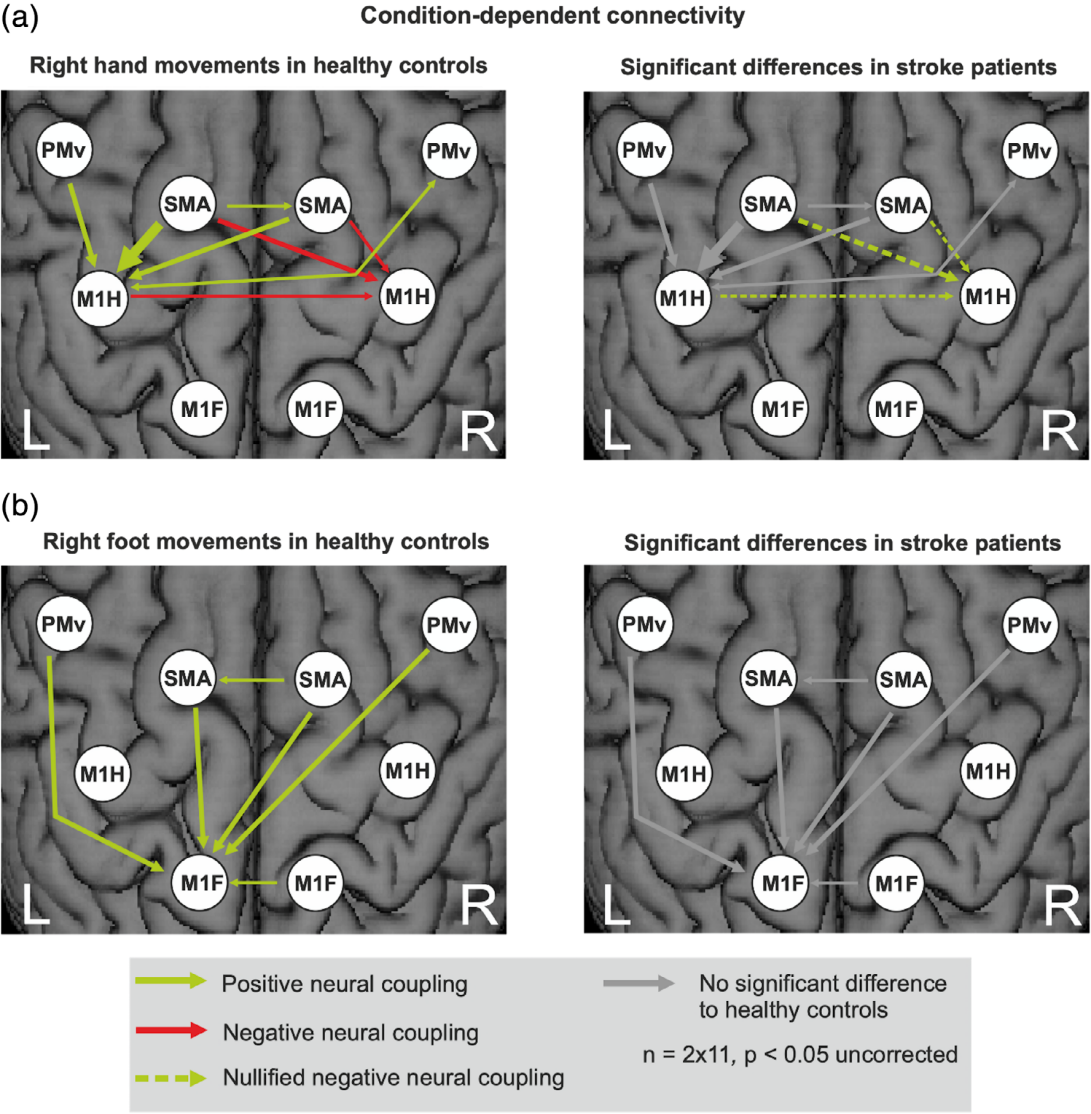
Condition‐dependent connectivity (dynamic causal modeling [DCM]‐B matrix) during unilateral movements. (a) Movements of the right hand of healthy controls and differences in stroke patients. (b) Movements of the right foot of healthy controls and differences in stroke patients. The width of each arrow corresponds to the coupling strength

Condition‐dependent coupling changes evoked by the experimental conditions, that is, unilateral movements of the right hand and foot, are represented by the DCM‐B matrix (Figure 8). Coupling parameters are quantified in Tables S2 to S5 of the Supplement.

Overall, connectivity changes evoked by limb movements were rather weak, probably owing to the reduced power of the sparse sample fMRI technique. However, at an uncorrected level (*p* < .05), the pattern of significant coupling parameters was very consistent with those reported in previous DCM studies using a similar model and a continuous sampling fMRI technique (Grefkes, Nowak, et al., 2008; Rehme, Eickhoff, et al., 2011; Volz, Eickhoff, et al., 2015). As this consistency across different studies with independent samples makes it rather unlikely that the effects were false positive observations, we report DCM‐B effects at an uncorrected level (*p* < .05). In healthy subjects, excitatory intrahemispheric and interhemispheric influences from premotor areas onto the active left M1_hand_, and inhibition from bilateral SMA onto the inactive right M1_hand_ were observed during movements of the right hand. In patients, the interhemispheric inhibition targeting M1 ipsilateral to the stroke‐affected hand was significantly weaker compared to healthy controls (Figure 8a, dashed arrows in right panel).

During movements of the right foot, bilateral premotor areas exhibited an excitatory influence onto left M1_foot_ in healthy controls (Figure 8b). No significant differences were observed between patients and controls during foot movements. Thus, only hand movements showed significant differences in coupling strength after stroke, while movements of the foot evoked a similar pattern of connectivity in both healthy subjects and stroke patients.

#### 3.3.4 Summary of findings

We here investigated a small sample of left‐hemispheric chronic stroke patients compared to an age‐matched healthy control group in a joint fMRI paradigm of hand and foot movements. Albeit well‐recovered, the patient group's motor performance was still significantly worse on their affected side (Figure 2) and led to an increased BOLD‐activity when moving the stroke‐affected hand or foot (Figure 3). For right hand movements this over‐activity was left lateralized, while right foot movements resulted in more bilateral (over) activations. Differential effects were observed depending on the motor effector: especially regions within the M1 representations of the affected limb showed significantly stronger increases in BOLD activity compared to healthy controls and compared to the respective other limb (interaction contrast, Figure 4), pointing to limb‐specific reorganization effects in M1. Importantly, changes in over‐activity in both the ipsi‐ and contralateral hemispheres were significantly correlated with worse behavior, implying a functional relevance (Figure 5). Endogenous connectivity showed a significantly weaker positive neural coupling from left SMA onto left M1_hand_ (that was also associated with worse motor performance) as well as a weaker interhemispheric coupling between left and right M1_foot_ in the patients relative to the healthy controls (Figure 7b). Task‐dependent connectivity (Figure 8) revealed that in stroke patients, interhemispheric inhibition was especially attenuated at the level of the hand motor area, which means that the known negative neural coupling between the movement related active (left) and inactive (right) M1_hand_ was missing. In contrast, at the level of the foot motor area connectivity was facilitatory in both groups, that is, during movement the right M1_foot_ exerted a positive influence on the left M1_foot_. In general, whereas intrahemispheric and interhemispheric inhibition targeting the inactive M1 was predominant during hand movements in our healthy subjects and missing after stroke, positive neural coupling onto the active M1_foot_ occurred during foot movements without being altered in our patient group, suggesting differential network reorganization effects underlying recovery of the upper and lower limbs.

## 4 DISCUSSION

The present pilot study aimed at gaining insights into the neural reorganization patterns of lower limb motor function in comparison to upper limb function in well‐recovered chronic stroke patients compared to healthy participants using fMRI and DCM. For the first time, we investigated unilateral movements of both the hands and feet in the same experimental setup using very similar paradigms. This enabled direct comparisons of the respective reorganization patterns underlying hand and foot movements as well as differential conclusions regarding their functional relevance.

### 4.1 Cortical reorganization underlying upper limb motion in chronic stroke

The finding of enhanced neural activity in stroke patients moving their paretic hand has long been reported by neuroimaging studies (Chollet et al., 1991; Gerloff et al., 2006; Pool et al., 2018; Rossini, Calautti, Pauri, & Baron, 2003; Ward et al., 2003). We here replicated these findings as our stroke patients showed increased neural activation in M1 and premotor cortex of the lesioned hemisphere when moving their affected hand, compared to healthy controls. Despite the consistent reports of over‐activation, its functional role remains controversial, especially regarding the contribution of the contralesional hemisphere (Buetefisch, 2015; Grefkes & Fink, 2011; Xerri, Zennou‐Azogui, Sadlaoud, & Sauvajon, 2014). While some studies suggest a supportive influence (Bütefisch et al., 2005; Lotze et al., 2006) other reports provide evidence for a maladaptive role of the contralesional hemisphere on hand motor function in chronic stroke (Mansur et al., 2005; Nowak et al., 2008; Takeuchi, Chuma, Matsuo, Watanabe, & Ikoma, 2005). One explanation for these heterogeneous findings are time‐dependent changes of neural activity, with a gradual reduction of bihemispheric overactivity with increasing time poststroke (Calautti et al., 2010; Rehme, Fink, et al., 2011) and a reestablishment of more physiological, that is, lateralized activation patterns concomitant to better recovery of function (Calautti et al., 2001; Ward et al., 2003). In line with these findings, our group of well‐recovered chronic stroke patients featured a rather lateralized sensorimotor network when executing simple movements with their right (affected) hand. However, activity within the lesioned hemisphere was still characterized by higher levels of ipsilesional activity within left M1_hand_ and PMv (Figure 3). Furthermore, enhanced neural activity was correlated with lower finger tapping frequencies not only within the ipsilesional but notably also the contralesional hemisphere (Figure 5). This supports the notion that persistent overactivity at chronic stages indicates less favorable outcome for upper limb recovery (Grefkes & Fink, 2012; Rehme, Eickhoff, et al., 2011; Ward et al., 2003).

As the primary origin of the corticospinal tract, M1 generates descending motor activity and is hence critical for movements of the contralateral limbs (Dum & Strick, 2002). Accordingly, sufficient levels of activation of the ipsilesional M1 are known to be a prerequisite for good motor performance after stroke (Favre et al., 2014; Peters et al., 2018). Thus, successfully recovered patients typically feature higher levels of activation in ipsilesional M1 during unilateral upper limb movements compared to patients suffering from pronounced deficits (Rehme et al., 2012) and patients who activated the posterior primary motor cortex early had a better recovery of hand function (Loubinoux et al., 2007). In line with these findings, limb‐specific neural activation changes in stroke patients compared to healthy controls were found within ipsilesional M1 (Figure 4). This emphasizes the importance of the ipsilesional M1 for functional recovery in chronic stroke patients.

We here analyzed effective connectivity to further our insights into the mechanistic relevance of altered neural activation patterns within the cortical motor network. Compared to the control group, chronic stroke patients featured a widely unchanged DCM‐A matrix consistent with their overall good clinical outcome. However, intrahemispheric positive coupling from left SMA onto left M1_hand_ was significantly reduced. This finding nicely aligns with previous reports of reduced excitatory influences from SMA onto M1 within the ipsilesional hemisphere in the subacute to chronic phase after stroke (Grefkes, Nowak, et al., 2008; Sharma et al., 2009) and increased excitatory influences from SMA onto M1 within the ipsilesional hemisphere concomitantly to motor recovery (Rehme, Eickhoff, et al., 2011) as well as a positive modulation of this connection during motor execution in patients following stroke and rehabilitation as shown by Bajaj et al. (2015a). Besides effective connectivity, also structural connectivity of M1 and SMA as a priori ROIs within the ipsilesional hemisphere was found to be correlated with upper limb motor scores of the affected extremity (Peters et al., 2018). Accordingly, we found that higher coupling strength exerted from ipsilesional SMA onto ipsilesional M1 was associated with better hand motor performance providing further evidence for a supportive role of premotor areas as mentioned above.

Concerning interhemispheric coupling influences, differences between patients and healthy controls were observed during movements of the affected hand (DCM‐B matrix). Here, chronic stroke patients featured a clearly attenuated interhemispheric inhibition onto the contralesional (inactive) M1_hand_, in line with previous studies reporting disturbed interhemispheric connectivity (e.g., insufficient interhemispheric inhibition targeting the unaffected M1_hand_) between primary hand motor cortices after stroke (Rehme & Grefkes, 2013; Takeuchi, Oouchida, & Izumi, 2012; Volz, Sarfeld, et al., 2015). M1‐M1 interhemispheric structural connectivity was also found to be significantly correlated with gross manual dexterity of the affected upper extremity (Peters et al., 2018). The observation of a negative M1‐M1 coupling is well in line with several electrophysiological experiments using double‐pulse TMS experiments that describe inhibitory influences between both M1 hand areas (Duque et al., 2005; Ferbert et al., 1992).

### 4.2 Cortical reorganization underlying lower limb motion in chronic stroke

Activity patterns during movements of the affected right foot were similar to healthy controls and were distributed more bilaterally concerning sensorimotor areas compared to unilateral hand movements (Kapreli et al., 2006; Luft et al., 2002). Significant stronger BOLD activity in the patient group was found ipsilesionally within left M1_foot_ and premotor cortex including PMv and bilateral SMA (Figure 3). A shift in motor network activation from the contra‐ to the ipsilesional primary sensorimotor cortex from the subacute to the chronic stage has been observed in a longitudinal fMRI study for locomotor recovery in stroke patients (Kim et al., 2006). Thus, enhanced neural activity, not only ispi‐ but also contralesional within left M1_foot_, left SMA as well as right PMv (Figure 5), seems to be functionally relevant and was found to be associated with greater motor impairment (i.e., lower tapping frequencies of the affected right foot) as measured outside the scanner. In line with our findings, Enzinger et al. reported increased cortical activation with increasing functional impairment (Enzinger et al., 2008). Consistent with studies in movement of paretic upper limbs, increased activation was observed in the contralesional hemisphere (primary sensorimotor cortex and SMA) during an ankle dorsiflexion fMRI. Unlike our results, no activation‐behavior correlation was mentioned for PMv. Differences in activation might be found due to slightly different motor tasks (ankle dorsiflexion vs. foot flexion including the toes in our case) as well as due to differences in clinical impairment. While Enzinger et al. included patients with residual gait impairments that had an MI for the affected leg of 77.7 (10.5), our patient group showed a higher degree of functional recovery with an MI of 90.3 (15.7). Thus, the contralesional hemisphere might have been less involved (Calautti et al., 2007; Loubinoux, 2003). Limb‐specific neural activation in stroke patients compared to healthy controls was found within left M1_foot_, that is, the respective ipsilesional primary motor cortex was significantly stronger activated during right foot than hand movements in patients. This corresponds to the results as mentioned earlier for the upper limb and underlines the symmetric albeit somatotopically structured reorganization pattern in functional recovery of the upper and lower limbs in chronic stroke patients. Our finding furthermore extends the importance of the ipsilesional primary motor cortical activity for upper limb recovery to M1_foot_ and lower limb recovery after stroke (Favre et al., 2014). Likewise, Peters et al. found the ipsilesional cortical disconnection of M1 on a structural level to be an independent predictor of motor performance, not only regarding the motor function of the affected upper extremity but also gait speed (Peters et al., 2018).

Furthermore, connectivity analyses revealed a positive interaction between left and right M1_foot_ representation. No additional interhemispheric inhibition was found between the primary foot motor cortices or from premotor regions. In contrast, stroke patients and healthy controls displayed significant positive interhemispheric influences between right and left M1_foot_ (DCM‐A matrix) and onto the left (active) M1_foot_ (DCM‐B matrix). The DCM‐A matrix additionally revealed negative interhemispheric influences between ipsilesional M1_hand_ and contralesional M1_foot_ that were significantly stronger in patients compared to healthy controls. This striking difference in interhemispheric coupling between movements of the upper (negative coupling) and lower limb (positive coupling) replicates findings previously published for a group of young healthy subjects (Volz, Eickhoff, et al., 2015). Interestingly, the interhemispheric positive coupling between the M1_foot_ representations was significantly less pronounced in the patient compared to the healthy control group in the DCM‐A matrix, whereas—unlike during movements of the right (affected) hand—no group differences were detectable dependent on movements of the right (affected) foot. This might generalize the finding of a task‐dependent detection of reduced global network connectivity poststroke where in a previous study pedaling instead of paretic tapping was able to elicit altered functional connectivity (Vinehout et al., 2019). While the DCM‐B matrix hence seemed to be adjusted to physiological levels in our group of well‐recovered stroke patients, we still found a persistent disturbed interhemispheric coupling between left and right M1_foot_ regarding endogenous connectivity. Further studies are needed to replicate and clarify if this is a very sensitive and persistent finding after stroke and therefore might be specifically targeted with neuromodulative strategies.

Similar to the results of Volz, Eickhoff, et al. (2015) and Volz, Sarfeld, et al. (2015), no significant inhibition of the contralesional (inactive) M1_foot_ by premotor areas was observed (Figure 8). Whereas stroke patients featured significant changes regarding premotor influences onto the contralesional right M1_hand_ during right‐hand movements, no changes between patients and healthy controls were found during movements of the affected right foot. Additionally, the functionally important excitatory influence from left SMA onto left M1_hand_ was not present for M1_foot_. Interestingly, the structural connectivity of the ipsilesional M1 and SMA mentioned above was not correlated with lower limb motor function, whereas anatomical connectivity between M1/SMA and the cerebral peduncle, thalamus and red nucleus was positively associated with the MI of the leg and gait speed (Peters et al., 2018). Thus, one reason for these limb‐specific differences might lie in a stronger impact of subcortical and spinal sources on lower limb function, associated with a weaker control at the cortical level, ultimately leading to differential cortical reorganization patterns underlying upper and lower limb motor function after chronic stroke (Jahn et al., 2008; Volz, Eickhoff, et al., 2015).

### 4.3 Limitations

Our cross‐sectional study offers first insights into limb‐specific changes in cortical reorganization, which need to be extended by longitudinal studies to establish a clear link between recovery and activation changes. Furthermore, a better characterization of the influence of different lesion locations on brain reorganization patterns seems mandatory. For example, in a study by Luft et al. (2005), unilateral knee movements led to differential cortical activation for the paretic and nonparetic leg dependent on lesions location (cortical, subcortical or within brainstem). Accordingly, the inclusion of additional regions (particularly subcortical) into the connectivity model would be of interest. However, the scope of the present study was to consider key regions of the cortical motor system, also given previous studies using similar models to allow comparisons between studies (e.g., Bajaj et al., 2015a, 2015b, 2016; Grefkes, Eickhoff, et al., 2008; Kim et al., 2018; Pool et al., 2018; Rehme, Eickhoff, et al., 2011; Volz, Eickhoff, et al., 2015; Volz, Sarfeld, et al., 2015; Wang et al., 2016).

Furthermore, we here investigated a preferably clinically homogeneous patient sample, which was, however, still heterogeneous concerning the lesion site, as lesions were located cortically, subcortically or both. Also, patients were not identically impaired in their paretic hand and foot motor function which is difficult to achieve in a clinical population. Finally, the overall sample size was too small to allow analyses of subgroups and comparison with the results mentioned above. We aimed at recruiting a homogeneous sample of stroke patients (i.e., first‐ever motor stroke, mild to moderate motor deficit, no concurrent other neurological deficit like aphasia or neglect, cognitively fit, no contraindications), resulting in a limited pool of suitable patients and ultimately a limited sample size. Potentially due to the limited sample size, the current results on condition‐dependent connectivity could only be presented at an uncorrected level and should, therefore, be interpreted with caution. However, previous studies using DCM in stroke patients have frequently used sample sizes between 10 and 15 patients and detected reliable effects in stroke patients, possibly since the effect sizes of stroke‐induced alterations in neural motor network dynamics are relatively large (Bajaj et al., 2016; Chu et al., 2018; Pool et al., 2018; Saleh et al., 2017; Schulz et al., 2016; Wang et al., 2016). Accordingly, we here found significant alterations and reasonable effect sizes for fMRI and DCM‐A as compared to our healthy control sample. Moreover, several of the group differences observed in the current dataset replicate earlier findings, corroborating the reliability of our findings (Grefkes, Nowak, et al., 2008; Rehme, Eickhoff, et al., 2011; Volz, Eickhoff, et al., 2015), also about DCM‐B. Ultimately, larger samples might yield higher sensitivity to discover further group differences of smaller effect size and might also be helpful to obtain stronger effects for connectivity analyses, especially from a “sparse sampling” fMRI design. As mentioned in the method section, a disadvantage of this method compared to a classical continuous‐sampling block design lies in its decreased statistical power due to the lower number of images per condition. Note, however, despite the weaker statistical power we considered a sparse‐sampling design more appropriate for the present study as this protocol minimizes movement‐associated artifacts during EPI acquisition. The latter is necessary when comparing motor tasks inducing different levels of head motion artifacts like hand versus foot movements (and which cannot be fully corrected by post hoc analyses; Seto et al., 2001; Weiss et al., 2013; Volz, Eickhoff, et al., 2015). By decoupling motor execution and EPI acquisition as achieved in the present study, it is more likely that the differences observed between limbs and groups are of neural origin and not induced by differences in movement artifacts (which typically induce the largest signal changes in EPI).

### 4.4 Conclusion and further implications

By investigating neural activity evoked by movements of the upper and lower limbs in one joint paradigm in healthy subjects and patients in the chronic phase after a stroke, we could observe commonalities as well as differences regarding cortical reorganization in terms of both changes in neural activity and effective connectivity. Especially interhemispheric connectivity at the level of M1 was differentially (re‐)organized after stroke, with disturbances of interhemispheric inhibition for hand movements and stronger facilitatory effects for movements of the feet. These exploratory findings may have interesting consequences for rehabilitation strategies aiming at improving motor outcome after stroke. For example, given the apparent differences in connectivity changes during hand and foot movements, it seems reasonable to hypothesize that interference with contralesional M1 activity via noninvasive brain stimulation, for example, applying inhibitory protocols, will probably exert differential network effects dependent upon whether the hand or foot representation is stimulated. Therefore, assessing differences in network changes for hands and feet through fMRI connectivity analyses might be useful to plan how to best interfere with brain activity to promote recovery of function after stroke.

## Supporting information


**Appendix** **S1**: Supporting Information

## Data Availability

The data are available from the corresponding authors upon reasonable request.
