# Supplementary material for: Cortical reorganization after motor stroke: A pilot study on differences between the upper and lower limbs
Source: Hum Brain Mapp. 2020 Nov 9;42(4):1013–33. doi: 10.1002/hbm.25275 (PMC7856649; doi:10.1002/hbm.25275)
Supplement: Supplementary file 1 — Appendix S1: Supporting Information [file HBM-42-1013-s001.docx]

**Supplement. Figure 1**

**Supplement. Figure 2**

Representative sections of maximum lesion overlap at the level of the corticospinal tract/posterior limb of internal capsule. Color shades represent the increasing number of overlapping lesions (N patients). Axial slices with the MNI-z-coordinates from 4 to 40 are shown. Individual lesion maps were smoothed with an isotropic FWHM kernel of 1 mm after spatial normalization to MNI space to account for variability in lesion delineation and warping.

| **Supplement.**  **Table I** |  |  |  |
| --- | --- | --- | --- |
| Endogenous connectivity in healthy controls (DCM-A). | | | |
| Significant coupling parameters are shown. | | | |
| **Connection** | **coupling parameters** | | ***SEM*** |
| SMA_L 🡺M1H_L | 0.150 | | *0.016* |
| SMA_R 🡺M1F_R | 0.112 | | *0.011* |
| SMA_R 🡺M1F_L | 0.096 | | *0.006* |
| PMv_R 🡺M1H_R | 0.085 | | *0.005* |
| SMA_L 🡺M1F_L | 0.082 | | *0.004* |
| PMv_R 🡺M1F_R | 0.072 | | *0.005* |
| PMv_L 🡺M1F_L | 0.065 | | *0.008* |
| SMA_L 🡺SMA_R | 0.044 | | *0.005* |
| SMA_L 🡺PMv_R | 0.044 | | *0.005* |
| SMA_L 🡺PMv_L | 0.043 | | *0.005* |
| SMA_R 🡺PMv_R | 0.040 | | *0.007* |
| SMA_R 🡺PMv_L | 0.040 | | *0.005* |
| SMA_L 🡺M1F_R | 0.039 | | *0.005* |
| SMA_R 🡺SMA_L | 0.035 | | *0.005* |
| PMv_R 🡺SMA_R | 0.035 | | *0.007* |
| PMv_R 🡺PMv_L | 0.034 | | *0.008* |
| PMv_R 🡺SMA_L | 0.032 | | *0.007* |
| M1F_L 🡺M1F_R | 0.030 | | *0.004* |
| M1F_R 🡺M1F_L | 0.030 | | *0.005* |
| PMv_L 🡺SMA_L | 0.027 | | *0.006* |
| PMv_L 🡺PMv_R | 0.026 | | *0.005* |
| M1F_L 🡺PMv_L | 0.025 | | *0.024* |
| M1F_L 🡺PMv_R | 0.024 | | *0.028* |
| M1F_L 🡺SMA_L | 0.024 | | *0.011* |
| PMv_L 🡺SMA_R | 0.024 | | *0.011* |
| M1F_R 🡺PMv_L | 0.023 | | *0.017* |
| M1H_L 🡺SMA_R | 0.023 | | *0.020* |
| M1F_R 🡺PMv_R | 0.022 | | *0.024* |
| M1F_R 🡺SMA_L | 0.022 | | *0.005* |
| M1F_L 🡺SMA_R | 0.021 | | *0.009* |
| M1H_L 🡺PMv_L | 0.019 | | *0.006* |
| M1F_R 🡺SMA_R | 0.019 | | *0.009* |
| M1H_L 🡺PMv_R | 0.016 | | *0.014* |
| M1H_R 🡺M1F_L | -0.024 | | *0.008* |
| L, Left; R, Right; | | | |
| M1F, primary motor cortex (M1) of the foot; M1H, M1 hand; | | | |
| SMA, supplementary motor area; | | |  |
| PMv, ventral premotor cortex; | |  |  |
| SEM, standard error of the mean | |  |  |

| **Supplement. Table II** |  |  |  |
| --- | --- | --- | --- |
| Endogenous connectivity (DCM-A). | | | |
| Significant differences in stroke patients. | | | |
| **Connection** | **coupling parameters** | | ***SEM*** |
| SMA_L 🡺M1H_L | 0.0660 | | *0.026* |
| M1F_R 🡺M1F_L | 0.0087 | | *0.004* |
| M1F_L 🡺M1F_R | 0.0085 | | *0.004* |
| M1F_R 🡺M1H_L | -0.0187 | | *0.005* |
| M1H_L 🡺M1F_R | -0.0262 | | *0.006* |

| **Supplement. Table III** |  |  |  |  | **Supplement. Table IV** |  |  |  |  |
| --- | --- | --- | --- | --- | --- | --- | --- | --- | --- |
| Condition-dependent connectivity (DCM-B). | | | | | Condition-dependent connectivity (DCM-B). | | | |  |
| Right hand movements in healthy controls. | | | | | Significant differences in stroke patients | | | | |
| Significant coupling parameters are shown. | | | | | (right hand movements). | |  |  |  |
| **Connection** | **coupling parameters** | | ***SEM*** |  | **Connection** | **coupling parameters** | | ***SEM*** |  |
| SMA_L 🡺M1H_L | 0.112 | | *0.014* |  | SMA_L 🡺M1H_R | 0.010 | | *0.008* |  |
| PMv_R 🡺M1H_L | 0.057 | | *0.021* |  | SMA_R 🡺M1H_R | 0.002 | | *0.005* |  |
| SMA_R 🡺M1H_L | 0.041 | | *0.018* |  | M1H_L 🡺M1H_R | 0.002 | | *0.002* |  |
| PMv_L 🡺M1H_L | 0.037 | | *0.013* |  |  | | |  |  |
| SMA_L 🡺SMA_R | 0.003 | | *0.001* |  |  | | |  |  |
| M1H_L 🡺PMv_R | 0.001 | | *0.000* |  |  |  |  |  |  |
| M1H_L 🡺M1H_R | -0.008 | | *0.002* |  |  | | |  |  |
| SMA_R 🡺M1H_R | -0.013 | | *0.005* |  |  |  |  |  |  |
| SMA_L 🡺M1H_R | -0.030 | | *0.012* |  |  |  |  |  |  |

| **Supplement.**  **Table V** |  |  |  |  |
| --- | --- | --- | --- | --- |
| Condition-dependent connectivity (DCM-B). | | | | |
| Right foot movements in healthy controls. | | | | |
| Significant coupling parameters are shown. | | | | |
| **Connection** | **coupling parameters** | | ***SEM*** |  |
| SMA_R 🡺M1F_L | 0.066 | | *0.013* |  |
| SMA_L 🡺M1F_L | 0.059 | | *0.013* |  |
| PMv_R 🡺M1F_L | 0.037 | | *0.013* |  |
| PMv_L 🡺M1F_L | 0.036 | | *0.010* |  |
| M1F_R 🡺M1F_L | 0.010 | | *0.002* |  |
| SMA_R 🡺SMA_L | 0.001 | | *0.000* |  |
